# Highly Parallel and Short-Acting Amplification with Locus-Specific Primers to Detect Single Nucleotide Polymorphisms by the DigiTag2 Assay

**DOI:** 10.1371/journal.pone.0029967

**Published:** 2012-01-13

**Authors:** Nao Nishida, Yoriko Mawatari, Megumi Sageshima, Katsushi Tokunaga

**Affiliations:** 1 Department of Human Genetics, Graduate School of Medicine, The University of Tokyo, Tokyo, Japan; 2 Research Center for Hepatitis and Immunology, National Center for Global Health and Medicine, Ichikawa, Japan; University of Navarra, Spain

## Abstract

The DigiTag2 assay enables analysis of a set of 96 SNPs using Kapa 2GFast HotStart DNA polymerase with a new protocol that has a total running time of about 7 hours, which is 6 hours shorter than the previous protocol. Quality parameters (conversion rate, call rate, reproducibility and concordance) were at the same levels as when genotype calls were acquired using the previous protocol. Multiplex PCR with 192 pairs of locus-specific primers was available for target preparation in the DigiTag2 assay without the optimization of reaction conditions, and quality parameters had the same levels as those acquired with 96-plex PCR. The locus-specific primers were able to achieve sufficient (concentration of target amplicon ≥5 nM) and specific (concentration of unexpected amplicons <2 nM) amplification within 2 hours, were also able to achieve detectable amplifications even when working in a 96-plex or 192-plex form. The improved DigiTag2 assay will be an efficient platform for screening an intermediate number of SNPs (tens to hundreds of sites) in the replication analysis after genome-wide association study. Moreover, highly parallel and short-acting amplification with locus-specific primers may thus facilitate widespread application to other PCR-based assays.

## Introduction

Polymerase chain reaction (PCR) is a commonly used technique in molecular biology. Several previously developed methods have employed multiplexed PCR in order to analyze genomic variations such as microsatellites or short tandem repeats (STRs), single nucleotide polymorphisms (SNPs) and insertions/deletions [1]–[3]. Multiplexed preparation of DNA templates in a single reaction is cost-effective, saving starting materials and run-time, while requiring careful optimization of assay conditions. The optimization process is highly empirical and time consuming, and depending on the combinations of markers, may or may not lead to successful assay development. For the conventional design of multiplex PCR, optimization of reaction conditions and careful pre-selection of targets are required in order to prevent excessive off-target priming by the numerous primers in the reaction. Moreover, the risk of generating errors in multiplex PCR, such as insufficient amplification, biased amplification and considerable primer-dimer formation within primers, tends to increase roughly as the square of the number of added primer pairs [4].

There are several approaches to resolving these drawbacks, including solid-phase assay formats (glass slide arrays, microbeads), oligonucleotides containing locked nucleic acid (LNA) residues and circularized amplification. Primers immobilized on the surface of the solid phase appear to markedly increase product yield on solid supports and may avoid the need for target pre-selection with a modification to enrich the input genomic DNA via a crude solution-phase multiplex PCR [5], [6]. LNA pentamers showed high priming efficiency to achieve small biased priming in multiplex PCR [7]. Circularized amplification avoids generating artifacts associated with conventional multiplex PCR where two primers are used for each target [8]. This procedure was shown to perform a 96-plex amplification of an arbitrary set of specific DNA sequences. The arrayed primer extension-based genotyping method (APEX-2) allows efficient homogeneous 640-plex DNA amplification with locus-specific primers [9]. These approaches show effective consequences for multiplex amplification, however, a small number of approaches are practically used in the field of molecular genetics, presumably due to its cost and time consuming steps in preparation.

We developed the DigiTag2 assay for multiplex SNP typing as a simple and cost effective approach by combining multiplex PCR to enrich genetic regions including the target SNPs and an oligonucleotide ligation assay to encode all of the SNP genotypes into well-designed oligonucleotides designated DNA coded numbers (DCNs) [10]. For an effective primer design for multiplex PCR, there are several important physical properties for primer sequences, including melting temperature, Gibbs energy of duplex between primer and template, and interactions between primers and PCR amplicons. The DNA polymerase enzyme used in a multiplex PCR is one of the important factors for a successful unbiased amplification.

The DigiTag2 assay is a suitable approach to analyze an intermediate number of SNPs (tens to hundreds of locus) in the replication study after genome wide association study [11]–[12]. However, the most time consuming step for the DigiTag2 assay in a total running time of 13 hours is multiplex PCR for target preparation (5.5 hours). Here, we report an improved protocol for the DigiTag2 assay with a short-acting multiplex PCR through the use of Kapa 2GFast HotStart DNA polymerase, which reduces total running time and increases assay throughput. In this study, we also validate the applicability of the 192-plex PCR with locus specific primers to amplify the target regions from genomic DNA, which leads to save genomic DNA samples.

## Methods

### DNA samples

Genomic DNA samples from 96 unrelated healthy donors were obtained from the Japan Health Science Research Resources Bank (Osaka, Japan). All donors provided written informed consent and samples were anonymized. One microgram of purified genomic DNA was dissolved in 100 µl of TE buffer (pH 8.0) (Wako, Osaka, Japan), followed by storage at −20°C until use.

### Primer design

A total of 192 pairs of primer were designed using the Visual OMP software version 7.1.0.0 (DNA software, Ann Arbor, MI, USA) with relatively long length (35–45-mer; average, 39.5-mer) to give amplicon sizes between 312 bp and 995 bp (average, 589 bp), each of which had an SNP site (Table S1). Prediction of DNA melting temperature was calculated using nearest-neighbor thermodynamic models. To avoid spurious amplification products, we employed a two-step protocol (denature and extension steps) using specifically designed primer pairs with an extension temperature at 68°C. The specificity of primer sequences was verified by Blat search in order to predict its location(s) on the human genome (GRCh37), and to confirm no unexpected SNP(s) within the primer sequence. The specificity of primer pairs was verified using MFE primer software, which can predict potential amplicon(s) generated from the human genome (GRCh37, up to 5 kb in amplicon size) [13]. All oligonucleotides (de-salted, 100 pmol/µl in TE (10 mM Tris-HCl, pH 8.0, 1 mM EDTA)) were purchased from Life Technologies (Carlsbad, CA, USA), and were stored at −20°C.

### Multiplex PCR with Kapa 2GFast HotStart DNA polymerase

Multiplex PCR mix had a final volume of 10 µl, including 10 ng of genomic DNA, 25 nM each primer, 1.5× KAPA2G Buffer (including 2.25 mM Mg^2+^), an additional 2.25 mM Mg^2+^ (final concentration of Mg^2+^: 4.5 mM), 0.2 mM dNTPs and 0.4 U of Kapa 2GFast HotStart DNA polymerase (Kapa Biosystems, Woburn, MA, USA). PCR amplification was conducted using a TGradient (Biometra, Göttingen, Germany) or PTC-225 (MJ Research, Waltham, MA, USA) as follows: 95°C for 3 min, followed by 40 cycles of 95°C for 15 s and 68°C for 2 min. When necessary, the fragment length of PCR products was confirmed by capillary electrophoresis (Agilent 2100 Bioanalyzer, Agilent, Santa Clara, CA, USA) in order to evaluate PCR efficiency. The total running times for multiplex PCR with Kapa 2GFast HotStart DNA polymerase using TGradient and PTC-225 were 1 h 48 min 55 s and 2 h 6 min 59 s, respectively.

### Multiplex PCR with QIAGEN Multiplex PCR Kit

Multiplex PCR mix had a final volume of 10 µl, including 10 ng of genomic DNA, 25 nM each primer, 1× Multiplex PCR Buffer (including 3.0 mM Mg^2+^), 0.2 mM dNTPs and HotStarTaq DNA polymerase (QIAGEN Multiplex PCR Kit; QIAGEN, Valencia, CA, USA). PCR amplification was conducted using a TGradient or PTC-225 as follows: 95°C for 15 min, followed by 40 cycles of 95°C for 30 s and 68°C for 6 min. The total running times for multiplex PCR with QIAGEN Multiplex PCR Kit using TGradient and PTC-225 were 5 h 27 min 53 s and 5 h 46 min 39 s, respectively.

### 96-plex genotyping by the DigiTag2 assay

**Figure 1:**
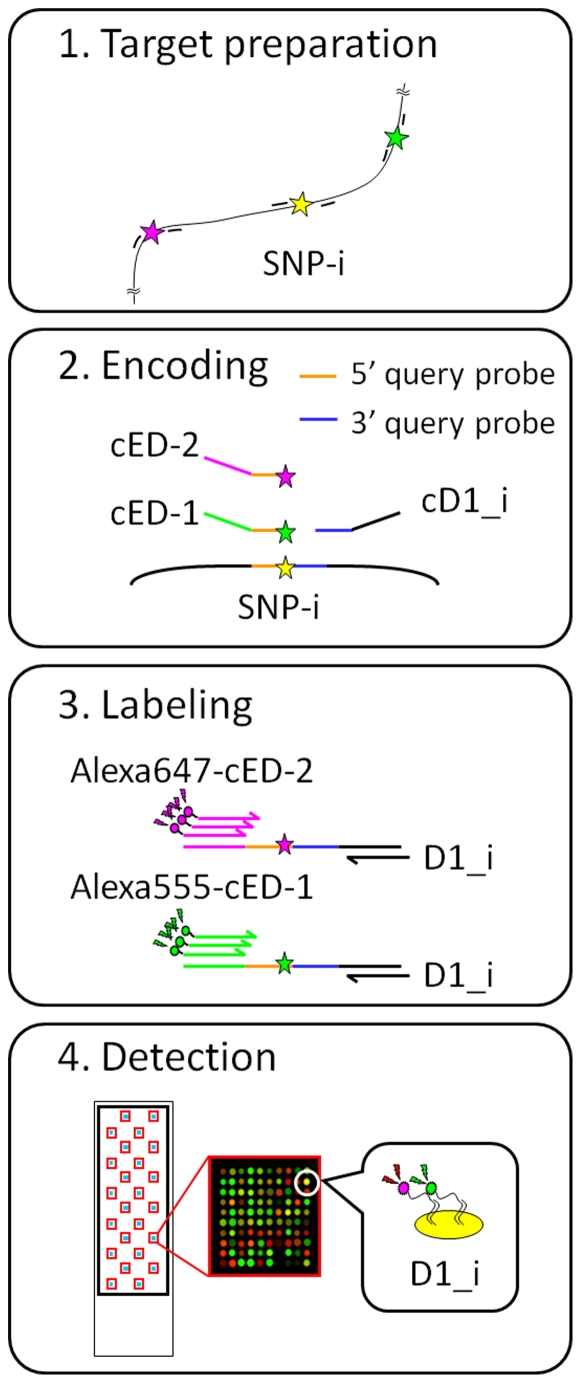
Schematic representation of the DigiTag2 assay. The assay has four steps: target preparation, encoding, labeling and detection. SNP genotypes are encoded into well-designed oligonucleotides, designated DNA coded numbers (DCNs: D1_i, ED-1 and ED-2). D1_i is a variable sequence assigned to each SNP. Reverse complement sequences are written by attaching the character ‘c’ before the sequence name.

The DigiTag2 assay performs multiplex SNP typing by encoding all of the SNP genotypes into well-designed oligonucleotides, designated DNA coded numbers (Figure 1, DCNs: D1_i, ED-1 and ED-2) [10]. The DCNs are assigned to the target SNPs in an unconstrained manner; therefore, the DNA chips prepared to read out the types of DCNs are universally available for any type of SNP without optimization of assay conditions. The DigiTag2 assay proceeds in four steps; target preparation, encoding, labeling and detection.

The encoding reactions had a final volume of 15 µl, including 0.5 µl of multiplex PCR products, 20 mM Tris-HCl, pH 7.6, 25 mM potassium acetate, 10 mM magnesium acetate, 10 mM DTT, 1 mM NAD, 0.1% Triton X-100 (1× Taq DNA ligase buffer) with 0.33 nM of each probe and 5 U Taq DNA ligase (New England BioLabs, Ipswich, MA, USA). Encoding reactions were conducted using a TGradient or PTC-225 under the following conditions: 95°C for 5 min, followed by 58°C for 15 min. The reaction was stopped by holding the temperature at 10°C.

The labeling reactions had a final volume of 12 µl, including 6 µl of ligation products, 0.5 µM each labeled primer (Alexa555-cED-1 and Alexa647-cED-2), 2.5 nM each D1 primer (D1_i), 50 mM KCl, 2 mM Mg^2+^, 0.1 mM DTT, 0.2 mM each dNTP (N = A, G, C), 0.1 mM [^3^H]-dTTP, 0.25 mg/ml activated salmon sperm DNA (1× *Ex Taq* Buffer) and 0.05 U of *Ex Taq*™ polymerase (TaKaRa, Shiga, Japan). Labeling reactions were conducted using a TGradient or PTC-225 under the following conditions: first held at 95°C for 1 min, followed by 30 cycles of 95°C for 30 s, 55°C for 6 min and 72°C for 30 s. The reaction was stopped by holding the temperature at 10°C. Total running times for labeling using TGradient and PTC-225 were 3 h 49 min 48 s and 4 h 8 min 48 s, respectively.

In the detection step, a hybridization mixture was prepared by mixing 6.25 µl of labeling products with 8.75 µl of hybridization buffer containing 0.5× SSC, 0.1% SDS, 15% formamide, 1 mM EDTA and 3.125 fmol of hybridization control (Alexa555-labeled D1_100 and Alexa647-labeled D1_100). The hybridization control was prepared for ensuring the hybridization step. Ten microliters of hybridization mixture was applied to each block on the universal DNA chip. Hybridization was carried out for 30 min at 37°C in a hybridization oven (ThermoStat plus; Eppendorf, Ham, Germany). After hybridization, glass slides were washed in washing buffer (0.1× SSC, 0.1% SDS) by shaking at 60 rpm for 3 min. Glass slides were consecutively washed in distilled water by shaking at 60 rpm for 1 min and then dried up by centrifugation at 500× g for 1 min. Hybridization images were scanned at photomultiplier voltages of 400 V for Alexa555 and 480 V for Alexa647 using a commercially available DNA chip scanner and fluorescence image analysis was performed using commercially available software (GenePix 4000B unit and GenePix Pro 4.1 software package; Molecular Devices, Sunnyvale, CA, USA).

### Labeling with Kapa 2GFast HotStart DNA polymerase

The labeling reactions with Kapa 2GFast HotStart DNA polymerase had a final volume of 12 µl, including 6 µl of ligation products, 0.5 µM each labeled primer (Alexa555-cED-1 and Alexa647-cED-2), 2.5 nM each D1 primer (D1_i), 1.5× KAPA2G Buffer (including 2.25 mM Mg^2+^), an additional 2.25 mM Mg^2+^ (final concentration of Mg^2+^: 4.5 mM), 0.2 mM dNTPs and 0.4 U of Kapa 2GFast HotStart DNA polymerase. Labeling reactions were conducted using a TGradient or PTC-225 under the following conditions: first held at 95°C for 1 min, followed by 30 cycles of 95°C for 15 s, 55°C for 120 s and 72°C for 5 s. The reaction was stopped by holding the temperature at 10°C. The total running times for labeling using TGradient and PTC-225 were 1 h 29 min 48 s and 1 h 48 min 34 s, respectively.

## Results

### Singleplex PCR using 192 pairs of locus-specific primers

**Figure 2:**
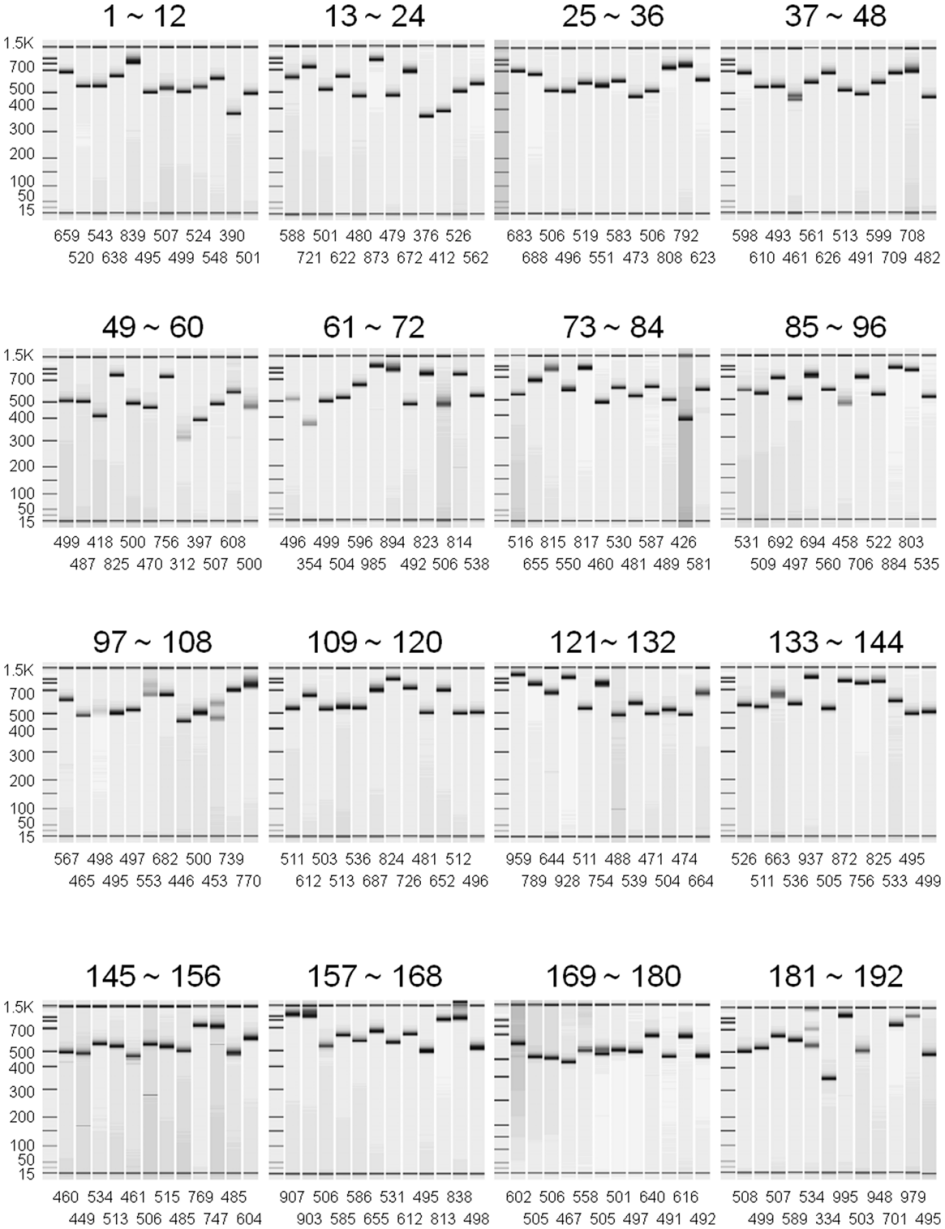
Electropherogram of singleplex PCR products with 192 pairs of locus-specific primers. The designed amplicon size is depicted below each lane.

Singleplex PCR was conducted under the same reaction condition with multiplex PCR using 25 ng of genomic DNA to ensure target amplicon detection and to confirm the emergence of extra bands (unexpected amplicons). Singleplex PCR with 192 pairs of locus-specific primers revealed that most of the primer pairs are able to achieve sensitive detection (concentration of target amplicon ≥5 nM) and specific amplification without extra bands (concentration of unexpected amplicons <2 nM) except for 14 pairs of primers; low sensitivity (<5 nM) for 5 pairs of primers (61, 99, 102, 189 and 191) and low specificity with extra bands (≥2 nM) for 9 pairs of primers (40, 56, 62, 70, 91, 106, 149, 173 and 174) (Figure 2 and Table S2). Five pairs among the 9 low-specific primer pairs with extra bands (62, 70, 149, 173 and 174) resulted from heteroduplex formation of target amplicons during polyacrylamide gel electrophoresis. Despite the presence of extra bands, the remaining 4 pairs of low-specific primers had a target amplicon with a detectable concentration ≥5 nM.

### Validation of efficacy of 192-plex PCR by 96-plex genotyping with the DigiTag2 assay

The DigiTag2 assay enables the simultaneous analysis of 96 target SNPs in: (1) multiplex PCR with locus-specific primers to amplify target genomic regions including target SNPs; (2) multiple oligonucleotide ligation assay with locus-specific probes to determine the genotype of each SNP; and (3) hybridization to the universal DNA chip tethered with probe sequences identical to D1_i (23-mer) (Figure 1) [10]. The validity of 192-plex PCR was assessed with 96 individual DNAs (population control samples) by comparing two sets of 96-plex genotype calls acquired from 96-plex PCR with those from 192-plex PCR (Table 1).

**Table 1:** Validation of efficacy of 192-plex PCR by 96-plex genotyping.

		192-plex PCR	96-plex PCR
1st set	Conversion rate	86/96 SNP	86/96 SNP
	Call rate	99.84% (7,728/7,740 genotype)	99.81% (6,695/6,708 genotype)
	reproducibility	99.99% (7,288/7,289 genotype)	100% (6,121/6,121 genotype)
	concordance	99.98% (6,289/6,290 genotype)	
2nd set	Conversion rate	87/96 SNP	87/96 SNP
	Call rate	99.79% (8,074/8,091 genotype)	99.79% (8,161/8,178 genotype)
	reproducibility	99.97% (7,792/7,794 genotype)	99.99% (7,712/7,713 genotype)
	concordance	99.97% (7,882/7,884 genotype)	

**Figure 3:**
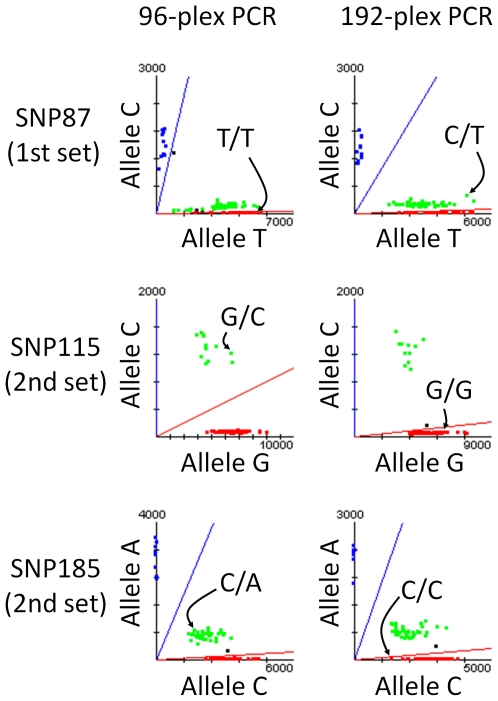
Scatter plots for three SNPs with 3 discordant genotypes. Scatter plots in genotyping with 192-plex PCR and 96-plex PCR are depicted side-by-side. The genotypes of discordant samples are indicated in the scatter plots by arrows.

Conversion rate shows the proportion of successfully genotyped SNPs with fewer than 3 undetected samples after excluding low-quality genotyping data, which had more than 5 undetected SNPs in a total of 96 SNPs. However, the composition of failed SNPs in genotyping was not identical, and the conversion rate showed no differences between 192-plex PCR and 96-plex PCR. For the 1st set of 96 SNPs, 7 SNPs among 10 failed SNPs were matched between 192-plex PCR and 96-plex PCR, and for the 2nd set, 8 SNPs among the 9 failed SNPs were matched. The average call rate for successfully genotyped SNPs was over 99.79% for both sets of 96-plex genotyping, even if 192-plex PCR products were adopted for target preparation. Reproducibility was determined by independent genotyping with 96 individuals twice. As a consequence, four discordant genotype calls were observed in the duplicated genotyping data. Concordance of genotype calls between 192-plex PCR and 96-plex PCR was determined using 6,290 genotype calls for the 1st set and 7,884 genotype calls for the 2nd set. Consequently, 14,171 out of 14,174 genotype calls were matched by comparison with 83 SNPs for the 1st set and 86 SNPs for the 2nd set. In total, 3 discordant genotype calls were observed (Figure 3).

### Short-acting multiplex PCR by use of Kapa 2GFast HotStart DNA polymerase

**Figure 4:**
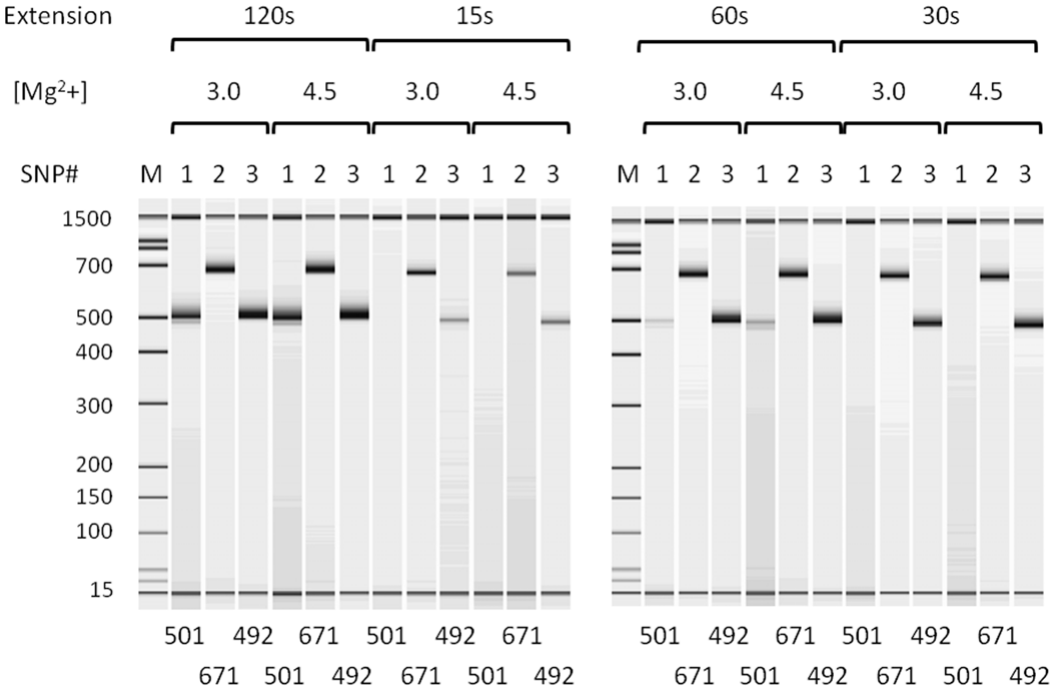
Electropherogram of singleplex PCR products using Kapa 2GFast HotStart DNA polymerase. Singleplex PCR was performed with varied extension periods (15 s, 30 s, 60 s and 120 s) and with varied Mg^2+^ concentrations (3.0 mM and 4.5 mM) using three pairs of locus-specific primers. The designed amplicon size is depicted below each lane.

Kapa 2GFast HotStart DNA polymerase was employed to perform multiplex PCR with the locus-specific primers for target preparation in genotyping with the DigiTag2 assay. To optimize reaction conditions with Kapa 2GFast HotStart DNA polymerase, singleplex PCR was conducted using 25 ng of genomic DNA with three randomly chosen pairs of locus-specific primers. The designed amplicon sizes for the three pairs of primers were 501 bp, 671 bp and 492 bp. We performed singleplex PCR using a two-step protocol (denature and extension steps) with varied extension periods (15 s, 30 s, 60 s and 120 s) and with varied Mg^2+^ concentrations (3.0 mM and 4.5 mM) (Figure 4). The most sensitive detection and highest levels of amplification for the three pairs of primers were observed with 120 s for the extension period and 4.5 mM for the Mg^2+^ concentration. The total running time for multiplex PCR with locus-specific primers was less than 2 hours, which is about 3 h 30 min shorter than the previous protocol (see MATERIALS AND METHODS).

The total running time of the DigiTag2 assay was markedly reduced when the labeling step was also conducted using Kapa 2GFast HotStart DNA polymerase instead of *Ex Taq* polymerase. When the DigiTag2 assay was conducted with Kapa 2GFast HotStart DNA polymerase for multiplex PCR and labeling step, the total running time of the assay was about 7 hours, which is about 6 hours shorter than the previously used protocol in combination with QIAGEN Multiplex PCR Kit for multiplex PCR and *Ex Taq* polymerase for the labeling step.

Table 2 summarizes the quality parameters (conversion rate, call rate, reproducibility and concordance) when genotyping was conducted with 192-plex PCR or 96-plex PCR by use of Kapa 2GFast HotStart DNA polymerase. The conversion rate was slightly decreased when multiplex PCR was conducted in 192-plex form. However, the conversion rates were better than those observed when multiplex PCR was conducted with the QIAGEN Multiplex PCR Kit. The composition of failed SNPs in genotyping was not consistent for the 1st set of 96 SNPs, in which 4 SNPs were matched between 192-plex PCR and 96-plex PCR. For the 2nd set, a total of 8 failed SNPs in the 96-plex PCR were completely matched to those in the 192-plex PCR. When the composition of failed SNPs were compared between Kapa 2GFast HotStart DNA polymerase and QIAGEN Multiplex PCR Kit, the 1st set had 5 matched SNPs in a total of 8 failed SNPs for 192-plex PCR, and 4 matched SNPs in 5 failed SNPs for 96-plex PCR. From the 2nd set, 5 SNPs in a total of 9 failed SNPs were matched when 192-plex PCR was conducted and 4 SNPs in a total of 8 failed SNPs were matched when 96-plex PCR was conducted. The average call rate for successfully genotyped SNPs was over 99.76% for both sets of 96-plex genotyping, even if 192-plex PCR products were adopted for target preparation. The reproducibility was 100% for the 2nd set; however, three discordant genotype calls were observed for the 1st set. With regard to the concordance of genotype calls between 96-plex PCR and 192-plex PCR, only one discordant genotype call was observed in the comparison for the 1st set, and no discordant genotype calls were observed in the 2nd set.

**Table 2:** Validation of efficacy of 192-plex and 96-plex PCR with Kapa 2GFast HotStart DNA polymerase.

		192-plex PCR	96-plex PCR
1st set	Conversion rate	88/96 SNP	91/96 SNP
	Call rate	99.84% (8,259/8,272 genotype)	99.76% (8,443/8,463 genotype)
	reproducibility	99.97% (8,069/8,071 genotype)	99.99% (8,339/8,340 genotype)
	concordance	99.99% (7,982/7,983 genotype)	
2nd set	Conversion rate	87/96 SNP	88/96 SNP
	Call rate	99.91% (8,171/8,178 genotype)	99.83% (8,346/8,360 genotype)
	reproducibility	100% (7,705/7,705 genotype)	100% (7,796/7,796 genotype)
	concordance	100% (8,161/8,161 genotype)	

Table 3 shows the concordance rate in comparison with the genotype calls by the use of Kapa 2GFast HotStart DNA polymerase or QIAGEN Multiplex PCR Kit for multiplex PCR. For the 1st set, there were 4 discordant genotype calls with 96-plex PCR and 8 discordant genotype calls with 192-plex PCR. For the 2nd set of 96 SNPs, there was one discordant genotype call in genotyping with 96-plex PCR and 192-plex PCR.

**Table 3:** Concordance of genotype calls between Kapa 2GFast HotStart DNA polymerase and QIAGEN Multiplex PCR Kit.

		Kapa 2G	QIAGEN
1st set	96-plex PCR	99.94% (6,513/6,517 genotype)	
	192-plex PCR	99.89% (7,441/7,449 genotype)	
2nd set	96-plex PCR	99.99% (7,778/7,779 genotype)	
	192-plex PCR	99.99% (7,700/7,701 genotype)	

## Discussion

The locus specific primers sufficiently worked in a multiplex form under the same reaction conditions without any optimization processes, either 96-plex PCR or 192-plex PCR. We also found that either 96-plex PCR or 192-plex PCR could be accomplished within two hours through the use of Kapa 2GFast HotStart DNA polymerase. The total running time of the DigiTag2 assay was shortened by 6 hours over the original 13-hour long protocol using Kapa 2GFast HotStart DNA polymerase for both multiplex PCR and the labeling step. The quality parameters (conversion rate, call rate, reproducibility and concordance) observed in genotyping with the new protocol were the same as those observed in the original protocol using QIAGEN Multiplex PCR Kit for multiplex PCR and *Ex Taq* polymerase for the labeling step. The DigiTag2 assay worked with a conversion rate of over 93.2% (179 / 192 SNPs), average call rate of over 99.80% (16,789/16,823 genotypes) and reproducibility of over 99.99% (16,135/16,136 genotypes) using 96-plex PCR under the new protocol. The composition of successfully genotyped SNPs was different when the genotype calls were acquired using the different polymerases (Kapa 2GFast HotStart DNA polymerase and QIAGEN Multiplex PCR Kit), which would result from a varying amplification bias in multiplex PCR. We also found that 192-plex PCR with locus-specific primers worked in 96-plex genotyping with the DigiTag2 assay, giving the same quality parameter data as those observed in genotyping with 96-plex PCR. However, the composition of successfully genotyped SNPs was not consistent between 192-plex PCR and 96-plex PCR, which may be explained by changing the interactions between primer pairs in 192-plex PCR and in 96-plex PCR. The composition of successful SNPs was not consistent when using different polymerases or multiplex systems in the multiplex PCR, which casts some shadows on the reliability of the assay. Regardless of the existing shadows, indeed, 96-plex and 192-plex PCR work with a high conversion rate in genotyping over 93.2%. To clear the existing shadows, it is necessary to continuously accumulate genotyping data.

In this study, fifteen discordant genotype calls were in total observed in the comparison of genotype calls with: i) duplicated genotyping data; ii) genotyping data by use of 192-plex PCR and 96-plex PCR; and iii) genotyping data with different types of polymerases (Table S3). Table S3 shows the genotype calls acquired 8 times under different conditions. All fifteen discordant genotype calls were analyzed with direct sequencing, of which 13 genotype calls were determined. In 8 of 15 discordant genotype calls, the genotype calls were completely different depending on the type of polymerase. The genotype calls acquired using Kapa 2GFast HotStart DNA polymerase were 100% concordant (6 of 6) with those acquired by direct sequencing. This suggests that SNP allelic bias in PCR amplification readily occurred with the QIAGEN Multiplex PCR Kit; however, the error rate in genotyping was only 0.04% (6 out of 14,886 genotypes). The remaining 7 discordant genotype calls were randomly observed in 1 out of 8 different conditions. This shows that the random error rates were almost equal in the genotype data acquired with both types of polymerases (4 out of 62,227 genotypes for QIAGEN Multiplex PCR Kit and 3 out of 66,008 genotypes for Kapa 2GFast HotStart DNA polymerase).

Among the five low-sensitivity primer pairs found on singleplex PCR (61, 99, 102, 189 and 191), no amplicons were detected by primer pair 189 and low concentrations (<5 nM) of amplicon were detected by the 4 other primer pairs (Table S2). Therefore, the SNP189 failed in genotyping, independently of the type of polymerase and multiplicity in multiplex PCR (192-plex or 96-plex). However, the SNP191, which was amplified by primer pair 191, was successfully genotyped only when the QIAGEN Multiplex PCR Kit was used for the multiplex PCR. The concentration of amplicon amplified by primer pair 99 was the same as the 2.8 nM observed with the amplicon amplified by primer pair 191. SNP99, which was amplified by primer pair 99, was successfully genotyped independently of polymerase type and multiplicity in multiplex PCR (192-plex or 96-plex). These results suggest that the sensitivity in genotyping with Kapa 2GFast HotStart DNA polymerase was lower than the previously used protocol with QIAGEN Multiplex PCR Kit. These results would be explained by a biased amplification with the shortened protocol using Kapa 2GFast HotStart DNA polymerase, which tends to lead to a consequent biased genotyping. However, the investigated number of primer pairs would not be sufficient to decide the sensitivity in genotyping; therefore, it is necessary to continuously accumulate genotyping data. As the investigated number of primer pairs was only 192 (384 primers) in this study, melting temperature of each primer and the number of potential amplicons predicted by the MFE primer software were strongly associated with low sensitivity and low specificity in an amplification, respectively (multiple regression analysis, *P* = 1.26×10^−37^ and *P* = 1.52×10^−21^, respectively).

Through the use of Kapa 2GFast HotStart DNA polymerase, the genotype calls for 96 SNPs can be acquired in about 7 hours by the DigiTag2 assay. The genotyping platform with high conversion rate plays an important role for the replication studies to identify the disease associated genes from candidate loci found in the GWAS (genome-wide association study). The DigiTag2 assay with an improved protocol will be an efficient platform for screening an intermediate number of SNPs (tens to hundreds of sites) in the replication studies. Because of limitations in the variation of DNA coded numbers (DCNs), 192-plex genotyping is not available for the current DigiTag2 assay. However, 192-plex PCR can save genomic DNA samples and time for target preparation. Moreover, 192-plex PCR is also available for direct-sequencing and other PCR-based assays to amplify the target regions from genomic DNA.

## Supporting Information

Table S1 **Sequence information of 192 pairs of locus specific primer.**
(XLSX)

Table S2 **Results of singleplex PCR with 192 pairs of locus specific primer.**
(XLSX)

Table S3 **The 15 discordant genotype calls in 8 different conditions.**
(XLSX)
